# Catheter ablation of epicardial premature ventricular complexes in patients with and without cardiac scar

**DOI:** 10.1007/s10840-026-02351-5

**Published:** 2026-05-23

**Authors:** Sania Jiwani, Kelly Arps, Muazzum Shah, Amrish Deshmukh, Jackson J. Liang, Rakesh Latchamsetty, Thomas Crawford, Krit Jongnarangsin, Hakan Oral, Fred Morady, Frank Bogun, Michael Ghannam

**Affiliations:** Division of Cardiovascular Medicine, Department of Electrophysiology, University of Michigan, Ann Arbor, USA

**Keywords:** Premature ventricular complexes, Magnetic resonance imaging, Ablation, Epicardial arrhythmia

## Abstract

**Background:**

The left ventricular epicardium is a common site of origin (SOO) among patients with idiopathic premature ventricular complexes (PVCs) undergoing catheter ablation procedures. Less is known about epicardial PVC ablation among patients with myocardial scarring. The objective of this paper is to report on the presence and impact of cardiac scar among patients with epicardial PVCs undergoing ablation procedures.

**Methods:**

In a retrospective analysis, patients with epicardial PVCs and late gadolinium enhanced cardiac magnetic resonance (LGE-CMR) imaging were included. Acute and long-term procedural outcomes were examined and stratified by the presence or absence of cardiac scar.

**Results:**

Twenty-nine patients were included (male 17/29(59%), age 55±14 years, ejection fraction 48±12%, PVC burden 25±12%, ischemic cardiomyopathy (CM) *n* = 3, non-ischemic CM *n *= 7, PVC induced CM *n* = 5). The SOO was left ventricular summit (*n* = 20), great cardiac vein (*n *= 2), cardiac crux (*n *= 4), or basal anterolateral epicardium (*n *= 3). Ablation within the CVS was limited by proximity to the coronary arteries (*n* = 16), inaccessibility of ablation catheter (*n* = 5), or elevated baseline impedance (*n* = 2). LGE-CMR scar was present at the arrhythmia SOO in 14/29(48%) patients. There were no differences in procedural, radiofrequency, or fluoroscopy times between patients with and without scar (*p* > 0.05). Ablation was successful in 14/29 patients and partially successful in 8/29 patients; the post-ablation PVC burden was 8±10% with no differences among those with or without cardiac scar (*P* > 0.05).

**Conclusion:**

Epicardial PVCs may be encountered in patients with and without structural heart disease, with LGE-CMR located at the PVC SOO in almost half of the patients. While LGE-CMR remains a powerful diagnostic modality, its utility in predicting procedural outcomes for epicardial PVC ablation may be limited.

**Summary:**

Among consecutive patients undergoing ablation of premature ventricular complexes with an epicardial origin, LGE-CMR defined scar was present at the site of origin in approximately 50% of patients.

## Introduction

Premature ventricular complexes (PVC) are a commonly encountered arrhythmia that can result in symptoms as well as the development of PVC-induced cardiomyopathy. PVCs with an epicardial site or origin have been shown to be associated with a higher risk of cardiomyopathy presumably due to worse mechanical dyssynchrony [1]. Catheter ablation of PVCs is a safe and effective treatment especially in patients in whom anti-arrhythmic drugs (AAD) are ineffective, not tolerated or not preferred for long-term use. However, catheter ablation of epicardial PVCs can be challenging due to anatomic barriers and is associated with suboptimal success rates [2]as well as delayed recovery of LV function [3]. Epicardial PVCs may be seen in patients with idiopathic arrhythmias [3]; the prevalence and significance of epicardial PVCs in patients with structural heart disease is unknown. The routine use of late gadolinium enhanced cardiac magnetic resonance imaging (LGE-CMR) may identify cardiac scars in patients with overt structural heart disease or apparently normal hearts [4], and may aid in procedural planning of PVC ablation. There is limited data on the utility of LGE-CMR for guiding catheter ablation of epicardial PVCs and its effect on outcomes. The purpose of this study is to report on patient and procedural findings among patients with epicardial PVCs undergoing ablation with routine pre-procedural LGE-CMR.

## Methods

This was a retrospective, single center study of consecutive patients who underwent catheter ablation of epicardial PVCs with preprocedural LGE-CMR imaging from October 2014 to July 2024. The study was approved by the Institutional Review Board at the University of Michigan.

### Cardiac magnetic resonance imaging

LGE-CMR studies were performed on a 1.5T scanner (1.5T Achieva Philips MR, Amsterdam, The Netherlands) with an 8-element phased array coil placed over the chest of patients in the supine position. Images were acquired with electrocardiography gating during breath holds. Ten to 15 min after intravenous administration of 0.1–0.15 mmol/kg of gadobenate dimeglumine (MultiHance; Bracco Diagnostics, Princeton, NJ), 2-dimensional late gadolinium-enhanced imaging was performed by an inversion recovery sequence (repetition time, 6.7 ms; echo time, 3.2 ms; in-plane spatial resolution, 1.4–2.2 mm; slice thickness, 8 mm) in the short-axis view and 3 long-axis views of the left ventricle. The inversion time (250–350 ms) was optimized to null the normal myocardium in the short axis and long axis of the left ventricle at matching cine image slice locations.

### Mapping and ablation

After written informed consent was obtained, multipolar catheters were inserted into the femoral vein and positioned in the high right atrium, the His bundle, and the right ventricular apex. Mapping was performed with a 3-dimensional mapping system (CARTO^®^; Biosense Webster, Inc, Diamond Bar, CA) and a force-sensing irrigated-tip ablation catheter (Thermocool^®^; Biosense Webster). A prespecified mapping and ablation approach was not used. PVCs originating in the left ventricle were mapped via a retrograde aortic approach after administration of heparin to obtain an activated clotting time > 250 s. Right ventricular PVCs were mapped after administration of 3000 units of heparin initially, followed by 1000 units every hour. Intracardiac electrograms were displayed on a recording system (EP-Workmate™; St. Jude Medical, Inc, St. Paul, MN) at a speed of 100 mm/s. Multichamber mapping including the right ventricle, left ventricle, and coronary venous system (CVS) was performed in all patients. Activation timing was measured from the onset of the local bipolar electrogram (EGM) to earliest onset of any PVC-QRS waveform on a 12-lead electrocardiogram. Pace mapping was performed using a pacing output of 10 mV and a pulse width of 2.0 ms. An epicardial SOO was identified when the earliest intracardiac bipolar EGMs were identified at sites within the CVS or epicardium, with > 10/12 lead EKG matching to the clinical PVC during pace mapping. Radiofrequency energy was delivered for 60 to 120 s at the earliest breakout site with normal saline as the catheter irrigant. Endocardial ablation was performed at 30–50 W for 60–120 s targeting a 10Ω impedance drop. For ablation within the CVS, angiography was performed to ensure adequate distance from the coronary arteries (> 5 mm) prior to ablation; starting power within the CVS was 10 W and titrated upwards.

Programmed ventricular stimulation was performed at the right ventricular apex and outflow tract using up to 4 extrastimuli at 3 different drive cycle lengths. If ventricular tachycardia (VT) was not induced, this was repeated during intravenous infusion of isoproterenol. A positive study was defined as induction of monomorphic VT lasting ≥ 30 s or necessitating termination due to hemodynamic compromise.

Acute procedural success was defined as complete elimination of the clinical PVC. Partial success was defined as either an > 80% reduction in the frequency without elimination of the clinical PVC or elimination of the clinical PVC without elimination of non-clinical PVC. Procedural failure was defined as < 80% reduction in PVC frequency at the completion of a 30-minute observation period. Long term success was defined as an 80% reduction during the follow up period.

### Statistical analysis

Continuous variables were expressed as mean ± 1 SD and were compared with the Student t-test. Discrete variables were compared by c^2^ analysis or with the Fisher exact test. A p value of < 0.05 was considered statistically significant. All statistical analysis was performed using R version 4.4.1 (R Foundation for Statistical Computing, Vienna, Austria).

## Results

### Baseline characteristics

Twenty nine patients undergoing catheter ablation of epicardial PVCs with preprocedural LGE-CMR available for review were included. The study population was comprised of 17/29 (59%) male patients with mean age 55 ± 14years (Table 1). The mean ejection fraction (EF) of the study population was 50 ± 11%. Fifteen patients (52%) had a known history cardiomyopathy (CM) including ischemic CM (*n* = 3), non-ischemic CM (*n* = 7), and PVC induced CM (*n* = 5). Two patients had implantable cardioverter defibrillators (ICD). The mean PVC burden prior to ablation was 27 ± 14%.

### Cardiac imaging

Scar was identified on LGE-CMR in 21/29 (72%) patients. Scarring was predominantly located in the endocardium (*n* = 2), epicardium (*n* = 7), intramurally (*n* = 5), or intramural with epicardial extensions(*n* = 7). LGE-CMR scar was present at the arrhythmia SOO in 14/29 (48%) patients, including 4/15 (27%) of patients without previously known CM. Out of the 14 patients with CMR scar at arrhythmia SOO, 12 (86%) patients had epicardial scar, and two patients had intramural scar in the immediate vicinity. The remaining 7 patients with scar not at the arrhythmia SOO were at site in different cardiac segments.

**Table 1:** Patient characteristics. Patient characteristics stratified by the presence or absence of late gadolinium enhanced cardiac magnetic resonance imaging defined scar at the arrhythmia site of origin

	Overall	LGE-CMR at SOO (+)	LGE-CMR at SOO (-)	p
n	29	15	14	
Age, years(sd)	53.14 (14.80)	51.60 (18.21)	54.79 (10.43)	0.572
Male Sex, n(%)	17 (58.6)	12 (80.0)	5 (35.7)	0.041
Pre-ablation EF, %(SD)	49.76 (11.36)	51.27 (12.54)	48.14 (10.16)	0.469
Post-ablation EF, %(SD)	52.21 (12.75)	55.36 (11.64)	49.54 (13.49)	0.274
Pre -ablation PVC burden, %(SD)	27.03 (13.71)	32.00 (14.89)	21.71 (10.37)	0.041
Post-Ablation PVC burden, %(SD	7.22 (10.83)	9.14 (12.64)	5.15 (8.48)	0.349
Cardiomyopathy, n(%)				0.186
None	14 (48.3)	10 (66.7)	4 (28.6)	
ICM	3 (10.3)	1 ( 6.7)	2 (14.3)	
NICM	7 (24.1)	3 (20.0)	4 (28.6)	
PVC Related	5 (17.2)	1 ( 6.7)	4 (28.6)	
Hypertension, n(%)	13 (48.1)	6 (42.9)	7 (53.8)	0.853
Hyperlipidemia, n(%)	12 (44.4)	5 (35.7)	7 (53.8)	0.576
Diabetes, n(%)	5 (18.5)	0 ( 0.0)	5 (38.5)	0.038
Atrial fibrillation, n(%)	1 ( 3.7)	1 ( 7.1)	0 ( 0.0)	1
CKD, n(%)	1 ( 3.7)	0 ( 0.0)	1 ( 7.7)	0.97
COPB, n(%)	2 ( 7.4)	2 (14.3)	0 ( 0.0)	0.496
Radiofrequency time, min(SD)	28.71 (17.68)	31.57 (18.46)	25.86 (17.06)	0.403
Fluoroscopy time, min(SD)	36.18 (18.85)	38.57 (14.72)	33.79 (22.56)	0.512
Procedural time, min(SD)	301.57 (91.04)	312.86 (96.93)	290.29 (86.87)	0.522

*LGE-CMR*, late gadolinium enhanced cardiac magnetic resonance imaging; *EF*, ejection fraction; *PVC*, premature ventricular complex; *ICM*, ischemic cardiomyopathy; *NICM*, non-ischemic cardiomyopathy; *CKD*, chronic kidney disease; *COPD*, chronic obstructive pulmonary disease

### Mapping and ablation

Twelve/29 (41%) patients had monofocal PVCs and 17/29 (59%) patients had multifocal PVCs during the procedure. The epicardial SOO of the predominant PVC was the left ventricular summit (*n* = 20), the anterior great cardiac vein (*n* = 2), the cardiac crux (*n* = 4), or the basal anterolateral epicardium (*n* = 3).

**Fig. 1:**
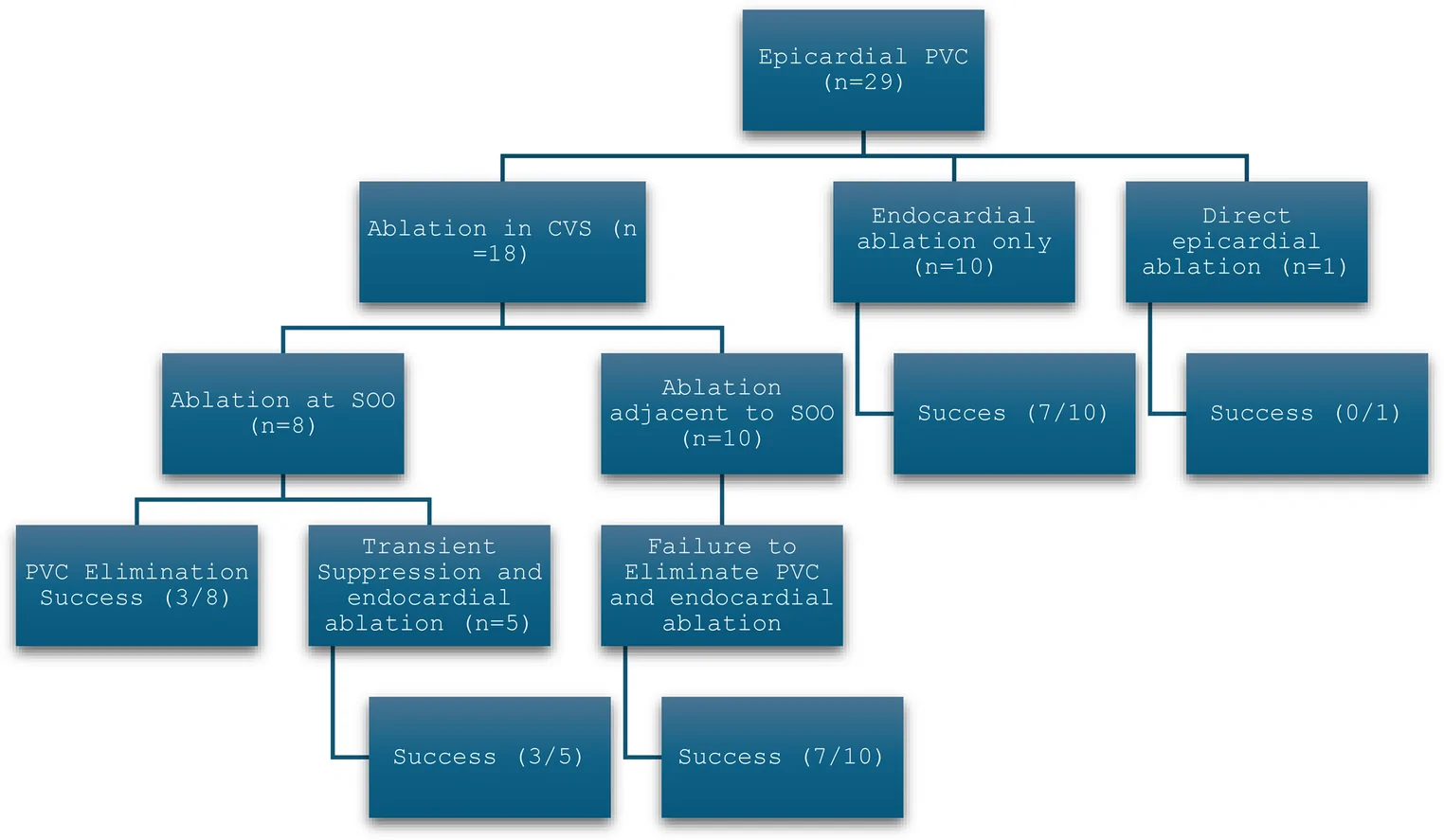
Procedural flowchart. PVC premature ventricular complex, SOO site of origin

**Fig. 2:**
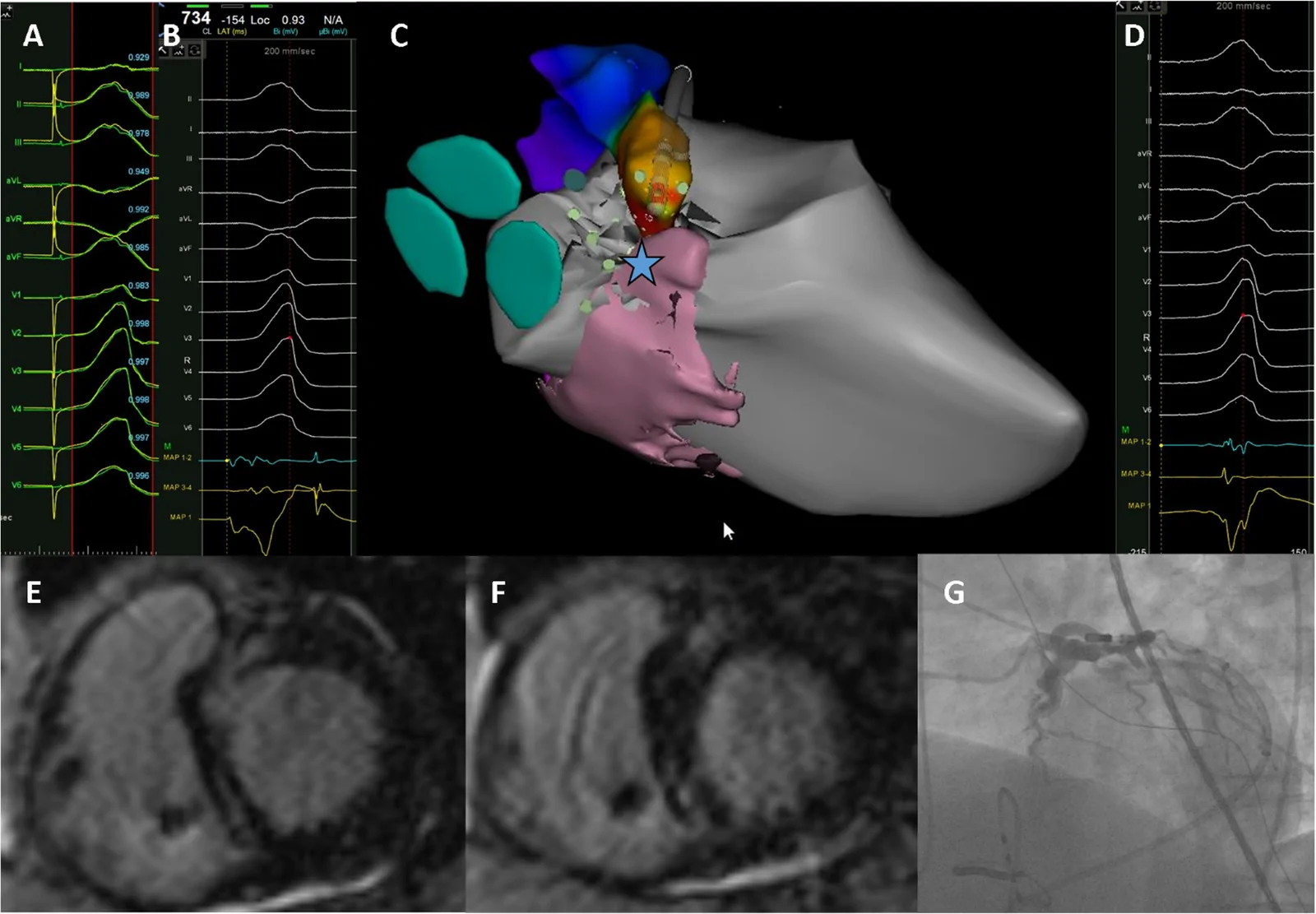
Ablation of epicardial PVC associated with cardiac scar. Panel **A** and **B**) Epicardial mapping was performed through instrumentation of the coronary venous system demonstrating 12/12 pace-mapping and early activation mapping located near at the left ventricular summit. Panel **C**) Three dimensional electroanatomic mapping is shown, with early activation within the coronary venous system (red gradient) overlaying areas of cardiac scar (pink) obtained from reconstructions of preprocedure late-gadolinium enhanced cardiac MRI (LGE-CMR). LGE-CMR imaging is shown in two short-axis reconstructions. Intramural cardiac scar is shown (Panel **E**) with extension to the epicardium (Panel **F**). Ablation at the site of origin within the CVS was limited by proximity to the left main coronary artery demonstrated on angiography (Panel **G**). Successful ablation was performed within the left ventricular outflow tract (Panel **C**, blue star), local activation time (Panel **D**) was late, however, ablation at this anatomically directed site which was overlying LGE-CMR scar tissue resulted in elimination of the clinical PVC

Ablation was performed in the coronary venous system (*n* = 18), left ventricular endocardium (*n* = 25), right ventricular endocardium (*n* = 4), coronary cusps (*n* = 7), and left ventricular epicardium (*n* = 1) (Fig. 1). All patients had matching pacemaps to the clinical PVC at the SOO, with an activation time of -26 ± 7ms preceding the PVC-QRS complex. Ablation was not performed in the CVS at the SOO in 11 patients, including 1 who underwent direct epicardial ablation via subxiphoid access. Among 10 other patients, ablation was limited due to proximity to the coronary arteries (*n* = 4), inability to advance the ablation catheter to the SOO (*n* = 5), or an elevated baseline impedance (*n* = 1) (Fig. 2). Among 18 patients who underwent ablation within the CVS, 10 patients were unable to undergo direct ablation at the SOO due to proximity to the coronary arteries. In these patients, the catheter was withdrawn and ablated at the site nearest to the epicardial SOO and remote from the coronary arteries. This failed to suppress the arrhythmia in all cases, and patients underwent ablation at additional LVOT (*n* = 9), RVOT (*n* = 3), and the left sinus of Valsalva (*n* = 3) sites. Ablation within the CVS at the SOO was performed in 8 patients, resulting in PVC eliminations (*n* = 3) or transient suppression (*n* = 5), with further ablation in the LVOT (*n* = 5) and aortic cusp (*n* = 1). Five patients developed chest pain during ablation within the CVS, despite ablation being performed >5 mm from the coronary arteries. In all patients, chest pain ceased upon discontinuation of ablation, no patients developed ST elevation and repeat angiography was unchanged.

Programmed stimulation was performed in 27 patients; 2/27 patients had inducible, sustained, monomorphic VT, both of which had LGE-CMR scar. There were no differences in procedural, radiofrequency, or fluoroscopy times between the two groups with and without cardiac scar on preprocedural MRI (*p* > 0.05).

### Complications

There were 3 procedural complications. Two patients developed cardiac tamponade requiring pericardial drain placement, both of which occurred as a result of catheter manipulation in the CVS. One patient developed a groin hematoma that resolved without intervention. There was no peri-procedural mortality.

### Procedural success and follow-up

The PVC ablation was deemed to be acutely successful in 15/29 (52%) patients and partially successful in 8/29 (27.58%) patients The acute success rate for the patients with SOO at the LV summit was 55% (11/20), at the crux it was 25% (1/4), at the basal anterolateral epicardium it was 33.33% (1/3), and at the anterior great cardiac vein it was 100% (2/2). The post-ablation PVC burden was 8 ± 10% with no differences among those with or without cardiac scar (*p* > 0.05). Among patients with acutely successful procedures, the post-ablation PVC burden was 4.3 ± 9.7%, among patients with partially successful procedures the PVC burden was 4.9 ± 3.8%, among patients with unsuccessful ablations the mean PVC burden was 15.6 ± 10.7%. Patients with summit PVCs had a pre-procedure PVC burden on 28.2 ± 9.9% and a post-procedure burden of 6.5 ± 9.9% (*P* < 0.05) and overall long-term success rate of 74%. One patient with a partially successful procedure with initial improvement in PVC burden had recurrence of PVCs and underwent a repeat catheter ablation procedure targeting PVCs of a different morphology. The repeat procedure was unsuccessful and complicated by pericardial effusion requiring pericardiocentesis.

Six patients remained on antiarrhythmic medications after the catheter ablation procedure (amiodarone *n* = 3, sotalol *n* = 2, propafenone *n* = 1). There was one death among the study population over a follow-up period of 3.2 ± 3.1 years secondary to traumatic intracranial hemorrhage.

## Discussion

### Main findings

In this series of consecutive patients undergoing ablation of epicardial PVCs with pre-procedure LGE-CMR imaging, structural heart disease was present in one half of patients. Epicardial PVCs were located primarily in perivalular regions including the cardiac summit, the crux, and the lateral mitral annulus, with evidence of LGE-CMR scar in almost half of the patients. Epicardial ablation at the site of origin was frequently limited by proximity to the coronary arteries requiring ablation at the surrounding cardiac chambers and left sinus of Valsalva in most patients. Procedural success rate was 72%, the most common complication was pericardial effusion due to perforation of the coronary venous system. Procedural metrics and success rates were comparable among patients with and without cardiac scar at the arrhythmia SOO.

### Epicardial PVCs and structural heart disease

An epicardial SOO is frequently encountered in patients with idiopathic PVCs [5]; less is known about the incidence of epicardial PVCs among patients with structural heart disease. The presence of structural heart disease may be underestimated without routine use of pre-procedure LGE-CMR, as cardiac scar may be seen in 25% of patients with normal EF undergoing ablation procedures [6]. Similarly, in this series of consecutive patients with epicardial PVCs and LGE-CMR, cardiac scar was seen in 48% of patients, including 27% without a prior history of cardiomyopathy. LGE-CMR scar has been shown to frequently colocalize to the arrhythmia SOO in intramural PVCs [7] and these results suggests a role in the arrhythmogenesis of focal epicardial VA as well.

Consistent with prior reports, the most common sites of epicardial PVCs were perivavlular locations including the cardiac summit and crux. The basal location of these arrhythmias affords an opportunity to perform mapping and ablation from within the CVS and aortic cusps. Frequently, the small caliber of the vessels and proximity to the coronary artery system limits direct ablation. Among 18 patients in whom ablation within the CVS was performed, only 11 were able to undergo direct ablation at the epicardial SOO. The remaining 7 patients underwent ablation both at the nearest possible CVS site remote from the true SOO, as well as within the ventricular endocardium and the aortic cusps targeting the anatomically nearest locations.

Strategies targeting summit PVCs have previously been explored including a variety of traditional and adjuvant approaches (such as bipolar ablation, alcohol ablation, or coronary angioplasty wire directed ablation) [8]. A subxiphoid approach has not been found to be beneficial in these patients predominantly due to the proximity of coronary arteries and the presence of epicardial fat [9]. An anatomic approach for targeting the SOO has been demonstrated to be feasible and safe and this is the currently preferred approach has been demonstrated [10, 11] given the frequent limitation of direct ablation of summit PVCs due to the proximity to the coronary arteries. Similarly, an anatomic approach was primarily used in this series with similar long-term success rates (74%) of prior studies. The use of adjuvant ablation techniques was not employed but can be considered for refractory cases.

LGE-CMR has emerged as an important modality in the management of ventricular arrhythmias, both in patients with and without known structural heart disease. LGE-CMR can reliably predict the optimal ablation approach in nonischemic cardiomyopathy, as purely endocardial or intramural scars are associated with endocardial ablation failure, while scars extending to the epicardium are amenable to successful epicardial ablation [12, 13]. Myocardial scar can also predict patient outcomes. The presence of myocardial scar has been found to be associated with a higher burden of PVCs and lower EF after catheter ablation [14]. Presence and burden of myocardial scar is predictive of future risk of VT and sudden cardiac death, independent of LV function [6, 15].

Our study findings showing no significant association between presence of scar on CMR and procedural characteristics and outcomes may be due to the limitations of current CMR imaging techniques for scar characterization. Additionally, anatomic barriers and proximity of the epicardial SOO to critical epicardial structures (e.g., coronary arteries) may impact the ablation strategy more than scar location alone. Operators may have to adapt procedural approaches based on electroanatomic and pace mapping along with anatomic challenges, irrespective of imaging findings. While LGE-CMR remains a powerful diagnostic modality, its utility in predicting procedural outcomes for epicardial PVC ablation may be limited. This study underscores the importance of integrating LGE-CMR findings with invasive mapping data, rather than relying solely on imaging to guide procedural strategy.

### Limitations

This was a retrospective, single-center study with a small patient population and these results should be confirmed in larger, multicenter studies utilizing predefined mapping and ablation approaches. High resolution LGE-CMR was not used which may have increased the detection of scarring in the area of interest. Adjuvant ablation techniques including ethanol ablation, simultaneous unipolar ablation, or bipolar ablation were not utilized which may have impacted on ablation outcomes. Post-ablation management of antiarrhythmics was performed at the discretion of the treating physician which may have influenced long-term outcomes.

### Conclusions

Epicardial PVCs may be encountered in patients with and without structural heart disease, with similar success rates among these two groups. Pre-procedure LGE-CMR demonstrated epicardial scar at the PVC SOO in almost half of the patients.

## Data Availability

No datasets were generated or analysed during the current study.
